# Trimethylamine-*N*-oxide switches from stabilizing nature: A mechanistic outlook through experimental techniques and molecular dynamics simulation

**DOI:** 10.1038/srep23656

**Published:** 2016-03-30

**Authors:** Anjeeta Rani, Abhilash Jayaraj, B. Jayaram, Venkatesu Pannuru

**Affiliations:** 1 Department of Chemistry, University of Delhi, Delhi–110 007, India; 2 Department of Chemistry, Indian Institute of Technology, New Delhi-110 016, India; 3 Supercomputing Facility for Bioinformatics & Computational Biology, Indian Institute of Technology, New Delhi-110 016, India; 4 Kusuma School of Biological Sciences, Indian Institute of Technology, New Delhi-110 016, India

## Abstract

In adaptation biology of the discovery of the intracellular osmolytes, the osmolytes are found to play a central role in cellular homeostasis and stress response. A number of models using these molecules are now poised to address a wide range of problems in biology. Here, a combination of biophysical measurements and molecular dynamics (MD) simulation method is used to examine the effect of trimethylamine-*N*-oxide (TMAO) on stem bromelain (BM) structure, stability and function. From the analysis of our results, we found that TMAO destabilizes BM hydrophobic pockets and active site as a result of concerted polar and non-polar interactions which is strongly evidenced by MD simulation carried out for 250 ns. This destabilization is enthalpically favourable at higher concentrations of TMAO while entropically unfavourable. However, to the best of our knowledge, the results constitute first detailed unambiguous proof of destabilizing effect of most commonly addressed TMAO on the interactions governing stability of BM and present plausible mechanism of protein unfolding by TMAO.

It is generally acknowledged that the functions carried out by the enzymes depend upon their structures as well as on the solutions in which they are found. In the last several years, a number of studies have elucidated the use of a group of low molecular weight compounds to reverse misfolded or mislocalized or aggregated forms of the proteins associated with the human diseases and also to refold the denatured proteins due to the various stresses^1^,^2^,^3^,^4^. In addition to having the versatility of general or specific affects, the use of strategy of these low molecular weight compounds i.e. osmolytes, is theoretically and practically attractive because of its applicability to a wide range of pathological conditions^1^,^2^,^3^,^4^,^5^,^6^. Notably, from the view point of basic biological and biomedical studies of the diseases caused by misfolding or unfolding of proteins, understanding the mechanism of protein folding/unfolding in presence of these osmolytes has become a major raised challenge.

Nature employs a variety of osmolytes such as polyols and sugars, amino acids and its derivatives and methylamines to cope with the osmotic stresses^6^,^7^,^8^. With reference to the biology of adaptation, trimethylamine-*N*-oxide (TMAO), a common osmolyte presents in large concentrations in the intracellular fluids of many species of all the kingdoms and mainly found in the tissues of marine elasmobranchs, has been well documented^9^,^10^,^11^. It has been suggested in the literature that TMAO, among the osmolytes, has received special interest because it has shown an extraordinary capability to reverse misfolded or mislocalized or aggregated or denatured proteins^9^,^10^,^11^. Although, there is no paucity of literature which highlight that TMAO is well known strong stabilizer for the majority of proteins studied^5^,^6^,^7^,^8^,^9^,^10^,^11^,^12^, it is not possible to give a clear unifying statement about the nature of TMAO against proteins.

Recent studies unveil the fact that TMAO can also behave as a denaturant which is intriguing general interest of the researchers^9^,^13^,^14^,^15^,^16^. According to Singh *et al.*^13^, TMAO is a destabilizer for lysozyme, RNase-A and apo-α-lactalbumin at pH below its pK_a_. Chilson and Chilson have also shown that at low pH, acid and guanidinium chloride denatured state of lactate dehydrogenase failed to refold in presence of TMAO^14^. Granata *et al.* reported that TMAO not only decreased thermal stability of prion protein (PrP) at low pH but behaved as a denaturant at room temperature^15^. Nandi and his co-workers observed the formation of the misfolded prion protein oligomers and their polymerization to amyloids in TMAO^16^. In addition to this puzzled area of research, the molecular mechanism of destabilization of protein in TMAO is a more complex problem and still unclear. This type of influence of TMAO on protein stability motivated experimental and theoretical groups to take a closer look at the molecular details of its interaction with the proteins.

To dissect the molecular basis of the action of TMAO on the protein, it is necessary to find out the answers of some questions, whether TMAO is directly affecting the protein or indirectly through water structure disturbances so that native basin of the protein would be destabilized. Despite the importance of the molecular understanding of interaction of TMAO with protein, it is a particularly daunting task. In shedding light on the mechanism of stabilization/destabilization of the protein in TMAO, is not a trivial question at all and this is the primary concern in the present study. The dearth of knowledge was really intriguing our interest in this research field. Our long range objective has been to uncover TMAO influence on the water structure nearby the protein, hydrated water structure of the protein and also whether preferential binding to the protein or exclusion from that.

Stem bromelain (BM) is a cysteine protease enzyme, a glycoprotein isolated from pineapple (Ananas comosus) which carries net positive charge at physiological pH. BM is 212 amino acid residues enzyme with molecular mass 23.8 kDa containing three disulphides and a single free cysteine (Cys) residue^17^,^18^. The various protease proteins in TMAO have been intensively studied from their chemico-physical and biological properties point of view and broadly used in a number of clinical, industrial and pharmaceutical applications^2^,^3^,^4^,^5^,^7^,^9^,^19^. It will be very interesting to explore whether all protease behaviors are consistent with the available literature. To find the answer to this question, BM was chosen to come across whether TMAO is a biocompatible co-solvent for its stability and activity. Moreover, a wide range of applications of BM in various fields is also the fact behind the choice of the system^17^,^18^. We also hypothesized that positive charge on BM surface is having great impact on the interaction of TMAO with the protein leading towards the stabilization/destabilization.

In present study, we investigate the influence of varying concentrations of TMAO on the conformational stability and activity of BM. We use fluorescence spectroscopy, circular dichroism (CD), UV-visible and Fourier transform infrared (FTIR) spectroscopy to explore these critical issues and to gain insight into the microscopic understanding of molecular mechanism of the protecting action of TMAO on BM. In addition, we combine these experiments with molecular dynamics (MD) simulation which is also an effective tool to obtain atomistic level framework for the understanding/delineating the interactions involved in the system of protein, TMAO and water. Consequently, the strategy can be successfully applied to design and synthesize BM in such a form possessing unusual stability against the changing environmental conditions including changes in the pH and temperature and also presence of denaturing agents. To the best of our knowledge, this study represents the first detailed experimental and simulation evidence about the mechanism of destabilization of protein by TMAO.

## Results

### TMAO-induced changes of thermodynamic parameters

The thermal unfolding curves of BM in varying concentrations of TMAO at pH 7 as compared to that in buffer are displayed in Supplementary Fig. 1. All the corresponding thermodynamic parameters from these thermal unfolding curves are collected in Table 1. Increasing concentration of TMAO till 1 M caused an increase in transition temperature (T_m_) as well as Gibbs free energy change of unfolding (**Δ**G_u_). The increased T_m_ values are accompanied by an increase in enthalpy change of unfolding (**Δ**H_m_) and entropy change of unfolding (**Δ**S_m_) at T_m_ (Table 1). These increases can be justified by respective explanation that there may increase in the intramolecular polar interactions in the folded state leading to more compact structure and decrease in the conformational entropy of folded state or increase in hydration entropy of denatured state^20^. In Table 1, the increase in heat capacity changes of unfolding (**Δ**C_p_) values at 25 °C is monitored upto 1M TMAO. It can be predicted here that a more compact native state is formed with less surface accessible surface area due to the conformational changes in the folded state in TMAO which leads to more increased SASA on unfolding^21^,^22^,^23^.

In Table 1, all T_m_, **Δ**G_u,_ **Δ**H_m_ and **Δ**S_m_ values are found to be decreased at higher concentrations of TMAO depicting more loose conformation of folded state. This may be the consequence of weakening of intramolecular polar interactions in the folded state and in turn, increased conformational entropy of folded state or decreased hydration entropy of the denatured state^20^. This increase in the concentrations of TMAO causes decrease in **Δ**C_p_ values at 25 °C indicating more open or more dynamic structure in the folded state so that a smaller increase is SASA on unfolding as compared to folded state (Table 1).

### Fluorescence detection of conformational changes in BM in the presence of TMAO

**Figure 1:**
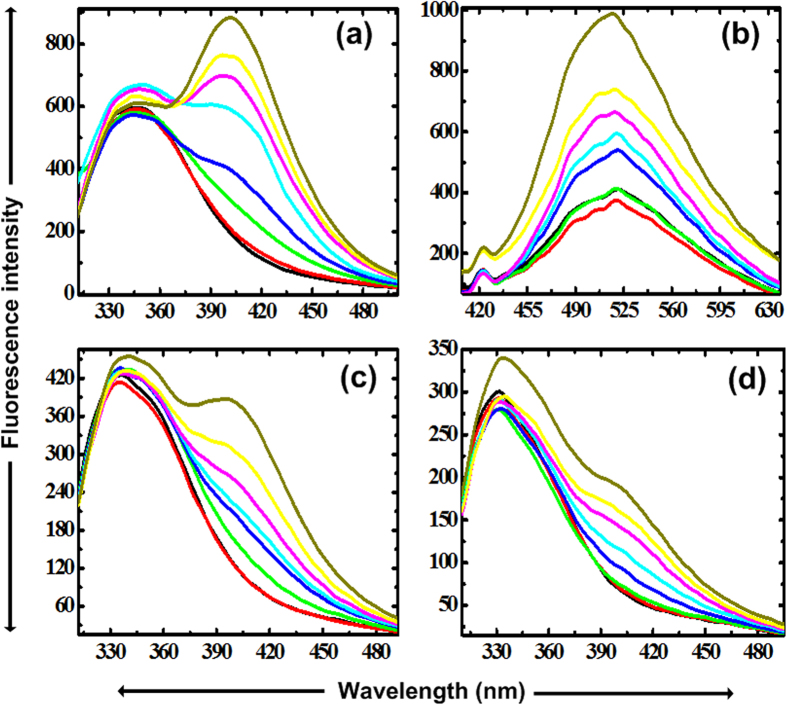
Fluorescence spectra analysis of BM at 25 °C in the presence of buffer (black) and varying concentrations of TMAO; 0.1 (red), 0.5 (green), 1.0 (blue), 1.5 (cyan), 2.0 (pink), 2.5 (yellow) and 3.0 M (dark yellow). (**a**) Trp fluorescence spectra upon excitation at 295 nm, (**b**) ANS fluorescence spectra upon excitation at 380, (**c**,**d**) Acrylamide quenching of Trp fluorescence spectra with acrylamide concentration 0.1 and 0.25 M, respectively, at an excitation wavelength of 295 nm.

The BM has five tryptophans (Trp) with two of them exposed to the surface^24^. Figure 1a corresponds to the Trp fluorescence spectra of the BM in absence and presence of the various concentrations of TMAO at 25 °C and pH 7. The BM in buffer shows wavelength maxima (λ_max_) at 347 nm. With increase in the concentration of TMAO upto 1 M, there is a slight decrease in intensity with no shift in λ_max_ as compared to the control. In addition, one another band around 400 nm is also started to appear which is noteworthy after 1 M TMAO. These two bands around 347 and 400 nm represent Trp of BM in highly contrasting environment as a function of TMAO concentration. This new band at 400 nm may be due to the contributions of the surface exposed Trp which must have been earlier highly quenched with no fluorescence due to the bounded water molecules and now has shown significant fluorescence with highly red shifted band. This red shift can be attributed to the replacement of water molecules nearby the exposed Trp by highly polar TMAO (4.92 Debye). The decreased intensity of the band at 347 nm may be due to the Trp-Trp energy transfer corresponding to the band at 400 nm. This type of Trp-Trp energy transfer is also consistent with earlier reports^25^,^26^. In addition to this Trp-Trp energy transfer, the decreased intensity for band at 347 nm can be due to the more compact structure as a function of TMAO concentrations till 1 M resulting in decreased distance between charged quenchers and buried Trp. For further increase in the concentration, there is the increase in the intensity with a red shift in λ_max_ from 347 nm. This may be caused by the loosening of the BM conformation which leads to the slight exposure of some of the buried Trp to the polar environment. However, the increase in the intensity at 400 nm can be attributed to more removal of the water molecules from exposed Trp^27^.

### 1-Anilinonaphthalene-8-sulphonate (ANS) binding to the BM in the presence of TMAO

Hydrophobic dye ANS has been widely used to detect the conformational changes in the protein as it monitors the exposure of hydrophobic surface in the protein during folding or unfolding. ANS binding to hydrophobic area emerges as a significant increase in the fluorescence emission of ANS^28^,^29^,^30^. ANS fluorescence emission in Fig. 1b represents two bands i.e. at 423 and 520 nm, for BM in buffer. The appearance of two bands indicates the presence of two ANS accessible hydrophobic pockets in BM. The hydrophobic pocket corresponding to the band at 423 nm may be in more interior of the BM and thereby less accessible for the ANS binding as is clear from very poor band in Fig. 1b. It is also unambiguous from Fig. 1b that this hydrophobic pocket is very less sensitive to the varying concentrations of the TMAO. On the other hand, hydrophobic pocket corresponding to the band at 520 nm is mainly accessible to the ANS binding which is responsible for the intense ANS fluorescence. At very lower concentration of TMAO, there is decrease in the fluorescence intensity with no or insignificant red shift in λ_max_. It emphasizes that BM structure is becoming more compact with internalization of hydrophobic groups as compared to the control. For further increase in the concentration of TMAO, there is the increase in the fluorescence intensity with blue shift which depicts the fact that there is more exposure of hydrophobic groups as a result of significant loosening of tertiary contacts in BM. All of a sudden, there is an emergence of highly intense fluorescence with blue shift in λ_max_ observed indicating large conformational changes towards unfolding of the BM at very higher concentration of TMAO (Fig. 1b). Additionally, it also reveals that BM is not completely denatured at this concentration as if any protein is fully denatured, there is no or loose binding of ANS resulting no fluorescence emission as proposed in earlier studies^26^,^28^,^29^.

### Acrylamide quenching studies for Trp fluorescence of BM in the presence of TMAO

In order to confirm whether newly appeared band at 400 nm in Fig. 1a is due to the surface exposed Trp, the conformational changes of BM in the presence of the varying concentrations of TMAO are also studied by the fluorescence quenching using acrylamide, a polar quencher^31^,^32^. Acrylamide is an effective and widely used quencher for fluorescence of either surface exposed or partially buried Trp^31^,^32^. Therefore, this phenomenon can be gainfully used to determine the accessibility of Trp residues. From Figs. 1c,d, it is clear that band at 400 nm which appeared at higher concentrations of TMAO as shown in Fig. 1a, is mainly affected upon addition of the acrylamide. Moreover, with increase in the concentration of acrylamide, more quenched Trp spectra are obtained for this band as can be seen in Fig. 1c,d. As a result, it can be emphasized that this band is mainly ascribed due to the contributions from the surface accessible and also from partially accessible Trp. The band at 333 nm is affected insignificantly which indicates that these Trp are more or less buried inside the protein core.

### CD spectroscopic analysis of conformation of BM in the presence of TMAO

**Figure 2:**
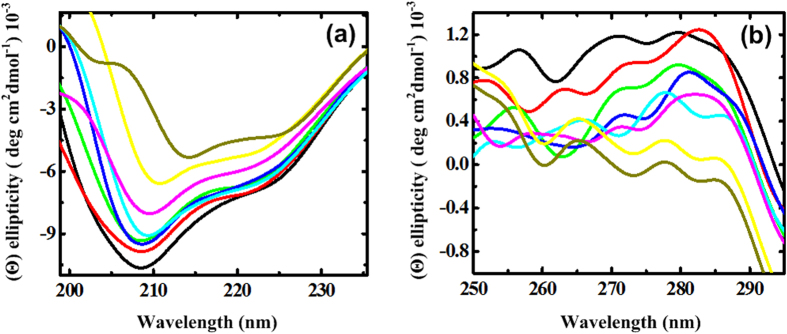
Influence of TMAO on the structure of BM at 25 °C from (**a**) far-UV CD analysis and (**b**) near-UV CD analysis: BM in the presence of buffer (black) and varying concentrations of TMAO; 0.1 (red), 0.5 (green), 1.0 (blue), 1.5 (cyan), 2.0 (pink), 2.5 (yellow) and 3.0 M (dark yellow).

Far-UV CD spectroscopy may provide qualitative information about the presence of secondary structural elements in the protein under the influence of the crowding agents. CD spectra are taken from range 200–250 nm because TMAO absorbs strongly below 200 nm. In Fig. 2a, a large negative band at 208 nm and a small negative band at 222 nm for the BM in buffer are known to be typical for protein containing α + β characteristics which is found to be consistent with report by Reyna *et al.*^33^. There is a decrease in the negative ellipticity of band at 208 nm with increase in the concentration of the TMAO as compared to the control. However, at very low concentration of TMAO, there is only a slight decrease in negative ellipticity of band at 208 nm indicating the secondary structures are more or less similar to the BM in buffer. At higher concentration of TMAO, there is a shift from α to β structures with overall reduction in α + β characters of the BM as is clear from Fig. 2a where with increase in the concentration of TMAO, band at 208 nm shifts to 214 nm which is a characteristic band for β sheets.

Figure 2b represents the near-UV CD spectra which reflect the environments of the aromatic amino acid residues (mainly Trp, Tyr and Phe) and thus gives information about the tertiary structure of the protein. As can be seen in Fig. 2b, there are CD bands around 256, 270 and 280 nm. The CD bands from individual residues may be positive or negative and may vary widely in intensity so it is often difficult to separate out the contributions of individual aromatic residues. Therefore, all spectra have been explained in a broad way concluding from overall changes in the positive ellipticity not particularly specifying the environment of three of aromatic amino acid residues (Trp, Tyr and Phe) and disulfide bonds. With increase in the concentration of TMAO, the observed decrease in positive ellipticities is implying, here, that the aromatic residues are moving towards less asymmetric environment as a result of loosening of some of the tertiary contacts.

### FTIR characterization of structural changes in BM in presence of TMAO

**Figure 3:**
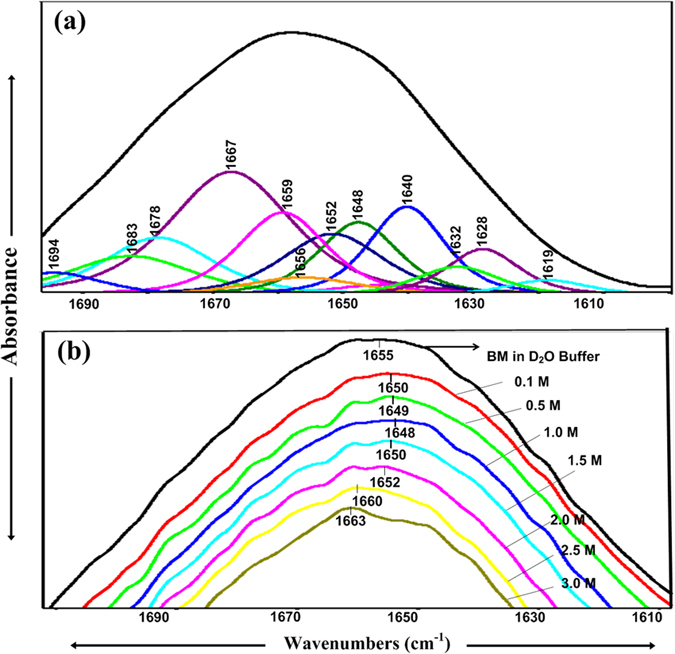
FTIR spectra analysis of BM. (**a**) FTIR curve fitted original spectrum of BM in D_2_O buffer at 25 °C where a smoothed original spectra is shown as black (**b**) Stacked FTIR original spectra of BM in the presence of D_2_O (black) and varying concentrations of TMAO; 0.1 (red), 0.5 (green), 1.0 (blue), 1.5 (cyan), 2.0 (pink), 2.5 (yellow) and 3.0 M (dark yellow).

Figure 3a represents amide I band in the range between 1610 and 1700 cm^−1^ for BM in D_2_O buffer at ~pD 7. The absorbances below 1620 cm^−1^are due to the side chain vibrations of aromatic groups as well as asymmetric stretching mode of COO^−^ groups which are ignored here as we are focusing mainly on secondary structure of BM. All components bands at 1628, 1632, 1640, 1647, 1651, 1656, 1659, 1667, 1678, 1683 and 1694 cm^−1^ are clearly observed in curve fitted original spectra of BM in buffer (Fig. 3a). The bands with very less integrated intensities are not shown in the Fig. 3a. The band assignment and total integrated intensity corresponding to the various secondary structures are shown in the Supplementary Table 1 and 2. Nevertheless, the obtained results are found to be in good agreement with the results reported elsewhere^33^. Further, the amide I region of all FTIR spectra in Fig. 3b are analyzed for secondary structure of BM as a function of TMAO concentrations. All the changes observed in the amide I maxima are related to the H-bond strength.

In Fig. 3b, the amide I band for BM appearing with a maximum at ~1655 cm^−1^ at 25 °C shifts to the lower wave number by ~7 cm^−1^ with addition of TMAO upto 1 M. Further, an increase in the concentration of TMAO more than 1 M leads to the shifting towards higher wavenumber. At 3 M of TMAO, band relocates to ~1663 cm^−1^. A shift towards the lower wavenumber may represent increased intramolecular H-bonding strength between the peptide bonds. The shifting towards the higher wave number may be attributed to the decrease in the intramolecular H-bonding in BM leading to the formation of loops, bends and other unordered structure. These types of observations are also reported in literature for various proteins^34^,^35^,^36^,^37^.

In addition, the band at ~2503 cm^−1^ corresponding to the O-D bond stretching is found to shift towards higher wavenumber by 3 cm^−1^ after an increase in TMAO concentration higher than 1M (Supplementary Fig. 2). However, band at ~1207 cm^−1^ for bending mode is observed to be shifted towards higher wavenumber by 4 cm^−1^ initially upto 1 M and after that there is a decrease in the bending frequency by approximately 4 cm^−1^ with increase in TMAO concentrations (Supplementary Fig. 3). These results indicate that water structure is disturbed at higher concentrations of TMAO as a consequence of decreased H-bonding in water molecules.

Apparently, total α + β characters are increased with increase in the concentration of TMAO till 1 M and that are decreased for further increase in the concentration of TMAO (Supplementary Table 2). It can also be emphasized that other structures comprising bends, loops and β-turns, are decreased as compensation. Some of the irregular structures are changed to the β-strand. Random coils again decreased with conversion of some structures into the helical structures except at 0.1 M where some of helical structures may be highly exposed to solvent, thereby, behaving as random coils.

As can be seen in Supplementary Table 2, for further increase in the concentration, there is an increase in the irregular structures (loops, bends and β-turns) as well as random coils and decrease in total α and β characters which may be resulting of loosed tertiary contacts. The irregular structures are increased to a large extent as a compensation of extended β-structures whereas there is high decrease in α-helical structures changing to the random coils. Even at higher concentrations of TMAO, BM is not a completely random coil. BM has three disulphide bonds that stabilize the globular structure of BM and TMAO will not disturb these tertiary contacts. As a result, BM structure is retained to some extent even at very high concentrations of strong denaturants^38^.

### Influence of TMAO on the activity of BM

**Figure 4:**
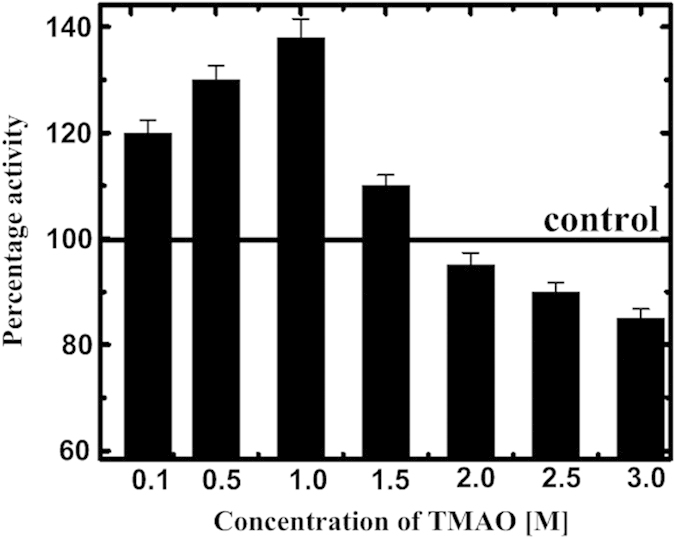
Proteolytic activity measurements of BM in the absence and presence of TMAO at 25 °C: the variation in percentage activity of BM in buffer (black control line) and in varying concentrations of TMAO; 0.1, 0.5, 1.0, 1.5, 2.0, 2.5 and 3.0 M. Error bars represent less than 5% error for three concordant measurements.

All the changes in the activity of BM in the presence of TMAO can be seen in Fig. 4. Initial increase in the caseinolytic activity of BM in TMAO till 1 M can be attributed to the conformational changes in the BM which leads to the decrease in the distance between the thiol group of active site residue Cys 25 and imidazole group of histidine (His 157) as compared to that in BM in buffer (~5 Å as reported in literature^39^,^40^ for BM under native conditions). For further increase in the concentration of TMAO, there is decrease in the activity, however, still higher than the control till 1.5 M. At very high concentration, activity is found to be decreased in comparison to control. The observed effects of TMAO on BM activity can be predicted in the way that the motion of active site region is significantly more constrained as compared to the other regions of the BM. The active site region remains partially undisturbed due to the disulphide linkages in BM.

### MD simulation studies of BM in the presence of TMAO

**Figure 5:**
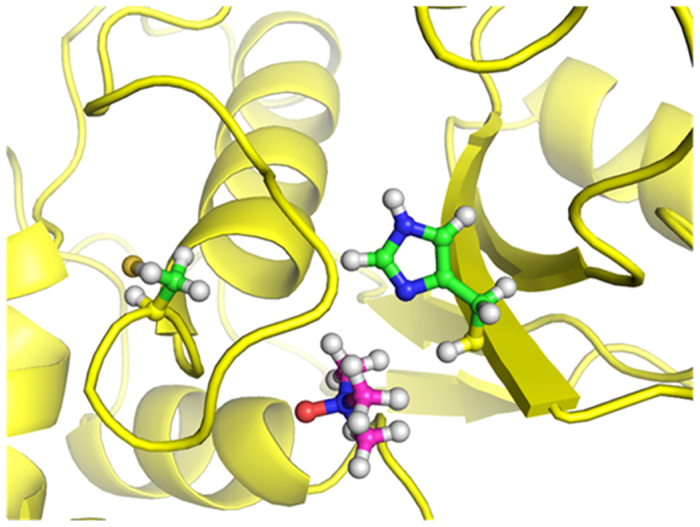
Partial conversion of catalytic cysteine containing helix region to loop in BM treated with 3 M TMAO. TMAO is represented as pink and catalytic residues in green.

**Figure 6:**
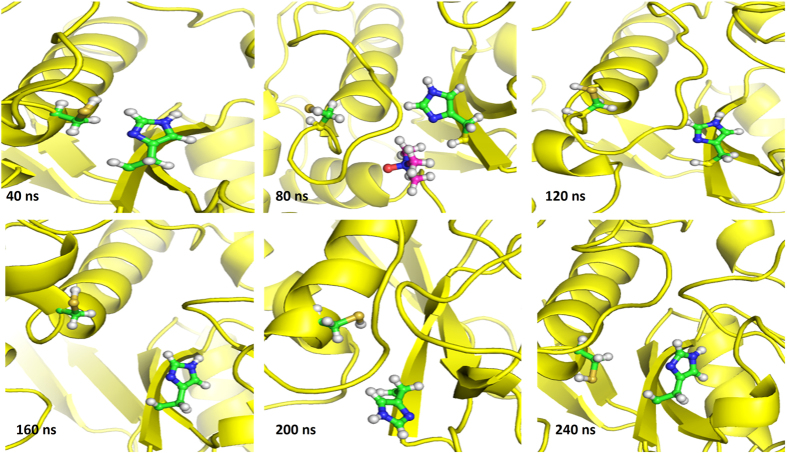
MD snapshots generated from 3 M TMAO treated BM after every 40 ns. Catalytic residues are shown in green and TMAO in red. The separation of the catalytic residues is shown to be induced by TMAO binding and conformational change of helix containing catalytic cysteine to loop.

**Figure 7:**
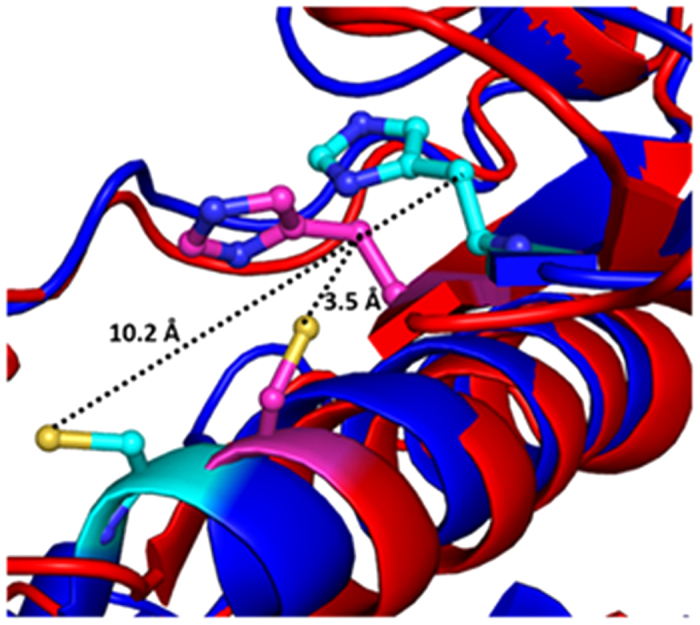
Distance between catalytic residues Cys 25 and His 157. BM treated with 3 M TMAO is represented as blue and cyan and native BM in red and pink.

**Figure 8:**
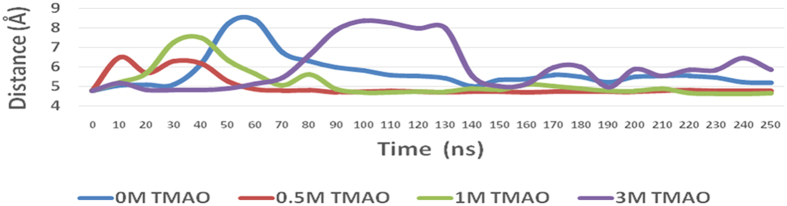
A plot of the distance between catalytic residues Cys 25 and His 157 as a function of simulation run length and as a function of concentration of TMAO (averaged over blocks of 10 ns).

It has been suggested that the thiol and imidazole groups of Cys 25 and His 157, respectively, act simultaneously in the hydrolysis of substrate by BM^39^,^40^. Hence the region around these residues has been studied for structural and conformational changes as a function of concentration of TMAO. Analysis of MD trajectories at high concentration of TMAO (3 M) reveals that the binding of TMAO near active site induces conformational changes near the catalytic residues (Cys 25 and His 157). The Cys 25 containing helix region is converted to loop along with a simultaneous increase in distance between the catalytic residues to >10 Å (Fig. 5). After the unbinding of TMAO, this loop region was found to revert back to helix while the distance between the catalytic residues remained >5 Å (Fig. 6) for most of the trajectory. The resultant separation of the two residues to a distance of 10.2 Å as compared to that in the native state i.e. <5 Å as can be seen clearly in the snapshot in Fig. 7. This increase in the distance leads to a decrease in the catalytic activity of BM. Events like this lead to continual destabilization of the conformations of these residues during the simulation. The distance variation between the two catalytic residues at different concentrations of TMAO is also illustrated in Fig. 8. The average distance between the catalytic residues was found to be 5.41 Å, 5.11 Å, 4.9 Å, 6.22 Å, for 0 M, 0.5 M, 1.0 M and 3 M TMAO, respectively. All these results are found to be well supported by the activity data using UV-vis spectroscopy where there is a decrease in the caseinolytic activity of BM at higher concentrations of TMAO (Fig. 4).

**Figure 9:**
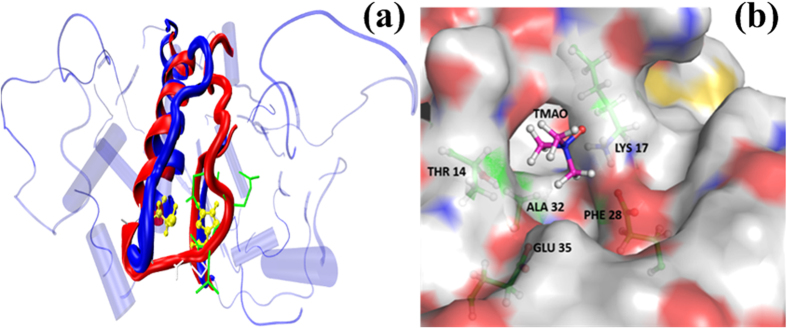
Loop movement of residues 15–20 forming part of the active site. (**a**) Untreated protein in blue and 3 M TMAO treated protein in red and (**b**) TMAO mediated stabilization of loop movement.

Additionally, TMAO induced changes in the loop conformation comprising residues 15–20 forming a part of the catalytic active site loop, are also observed in the simulation as can be seen in Fig. 9a. TMAO is found to interact preferentially with residues Thr 14, Lys 17, Ala 32, Phe 28 and Glu 35 of the pocket on BM (Fig. 9b). This, in turn, helps in stabilizing the altered conformation of loop region comprising residues 15–20. The altered loop conformation was attained after first 50 ns of simulation and was the preferred orientation for 80 percent of the rest of the period of 200 ns of the simulation. Minor loop movements were observed for 20 percent of the time but the loop comprising residues 15–20 did not revert back to its native conformation completely. The altered conformation is retained by the protein (BM) for most of the simulation showing its energetic favourability at 3 M TMAO. These changes in the conformation may lead to partial blockage of the catalytic active site, resulting in a loss of functionality of BM to some extent.

**Figure 10:**
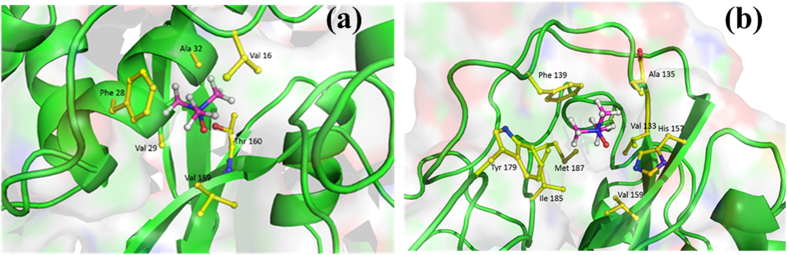
Interactions of TMAO with hydrophobic pockets of BM.

**Figure 11:**
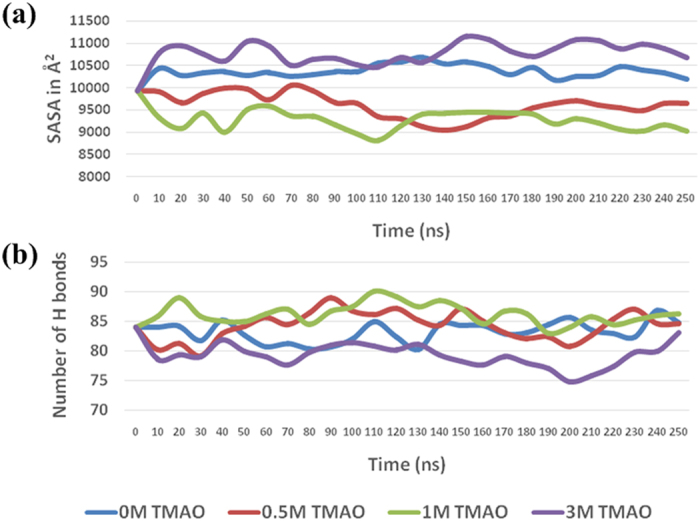
MD simulation analysis of BM. (**a**) Plot of BM SASA as a function of simulation run length and as a function of concentration of TMAO (averaged over blocks of 10 ns) and (**b**) Plot of number of hydrogen bonds within the BM as a function of simulation run length and as a function of concentration of TMAO (averaged over blocks of 10 ns).

Moreover, TMAO is found to interact with hydrophobic pockets in the BM at high concentrations as seen in Fig. 10a,b. These interactions were found to have an average lifetime of around 2 ns for the period of complete 250 ns and found to be 8 times more likely at higher concentration of 3 M TMAO compared to lower concentrations of 0.5 M and 1 M. This results in opening of hydrophobic pockets of BM which is captured by increased SASA and lowering of intra-molecular H-bonding as shown in Fig. 11a,b, respectively, at 3 M TMAO as compared to the control. TMAO is found to be preferentially excluded from the protein surface at lower concentrations resulting in a decreased SASA and increased intra-molecular H- bonding. On the other hand, TMAO gets preferentially accumulated at protein surface through polar/nonpolar interactions at high concentration of TMAO. The changes in SASA and H-bonding observed in molecular dynamics were confirmed with the data obtained from FTIR and ANS binding studies.

Therefore, it can be concluded that deviation in the backbone of BM at higher concentration of TMAO, mainly occurs in the area that is normally more susceptible to the motion such as active site loop. Other higher deviations may result from either direct H-bonding of TMAO with charged residues such as Lys and Arg or through concerted non-polar and polar interactions of TMAO with the residues in hydrophobic pocket of BM.

## Discussion

To probe into the mechanism behind the destabilization of BM in presence of TMAO is a really very difficult task and required a reasonable approach. Our study illustrates the power of integration of experimental and simulation methods which proved a well poised and a promising route to rise to this challenge. To make deep molecular basis analysis of all experimental and MD simulation results and reach upto the final aim of the manuscript, first lingering question is: Is pH is playing a vital role in destabilizing BM at higher concentrations of TMAO? We found that pH is not varying to a large extent with increasing concentration of TMAO and variation in the range of pH 7–8 is not influencing the stability and activity of BM as is reported by Ahmad *et al.*^41^. What nature of the protein compels TMAO for behaving against its well known nature i.e. natural stabilizer of the protein^42^,^43^? Is this inconsistent behaviour of TMAO presenting unusual and unresolved facts regarding preferential binding or exclusion?

To access the robustness of our results, there is a need of the consideration of two different cases for insertion of TMAO in the protein solution; in bulk that is far from the protein and in the hydration layer of the protein. TMAO is generally classified as a hydrophobic solute with propensity to partition to air/water interface despite its relatively high solubility in aqueous solution^44^,^45^. However, it is a well known fact that TMAO is strongly excluded from the peptide groups of protein, even though a mild attraction to the residue specific side chains may be present^45^,^46^,^47^. Surprisingly, according to some recent reports, its methyl groups are not strictly hydrophobic in nature due to the positive charge on the nitrogen, therefore, experience specific unfavourable interactions with the hydrophobic groups present on the surface of the protein^48^,^49^.

In addition, TMAO is neutral compound at physiological pH and its dipole moment is much larger than water molecule resulting strong H-bonding with water molecules as compared to water-water interactions and form di/trihydrated TMAO complexes^9^,^44^,^45^,^46^,^47^,^50^. Besides, the positive charge on the nitrogen also encourages hydrogen on methyl groups to form weak H-bonds to water molecules in aqueous solution^48^,^49^. Under diluted conditions, TMAO appears to interact with the peptide only through its hydration water^51^. Thus, hydrated TMAO complex is preferentially excluded from the surface of the BM resulting stabilization of BM at lower concentrations. The stabilization by TMAO can be easily examined from values of thermodynamic parameters in Table 1.

Moreover, at these concentrations, TMAO may start to withdraw water molecules even from the first hydration layer at BM surface as a function of increasing concentration which causes a reduction in the number of hydrogen bonds of water molecules with the protein backbone^21^. As a consequence, intramolecular interactions between different groups of the protein increases and have been examined by thermodynamic parameters (**Δ**H_m_ values in Table 1) indicating increased van der Waals interaction or other polar interaction in native form^20^,^52^,^53^,^54^,^55^ and also by FTIR results (Fig. 3b and Supplementary Table 2) which accentuate directly on sufficiently increased intramolecular hydrogen bonding in BM. MD simulation analysis also put emphasis on increased intramolecular H-bonding (Fig. 11b).

Furthermore, the increased thermal and conformational stability till 1 M i.e. increased T_m_ and **Δ**G_u_ (Table 1), also depict overall increased stabilizing forces which may also be ascribed to the increased van der Waals interactions, some polar interactions and hydrophobic effects. As a result of increased stabilizing interactions, a more compact native state with decreased SASA is formed at these lower concentrations of TMAO which leads to increased **Δ**S_m_ as well as **Δ**C_p_ for unfolding of BM (Table 1). These conclusions are entirely supported by the ANS binding and MD simulations results at these concentrations (Figs 1b and 11a). This compact structure must be mediated through some conformational changes that bring active site residues in more appropriate distances leading somewhat increased activity of BM (Figs 4, 5, 6, 7, 8). The compactness of the conformation of BM may also be the cause for bringing distinct Trp somewhat closer to each other in such an orientation resulting Trp-Trp energy transfer (Fig. 1a).

The structure of water in TMAO is quite different from that in TMAO in peptide or protein solutions. Three dimensional arrangements of water molecules around the oxygen of TMAO which acts as an acceptor for H-bonds, cause a long range geometric defect in the structure of the water at microscopic level in bulk^50^ and also near the hydration shell of the protein. This can be attributed to the asymmetric nature of the excess H-bond acceptor sites at the hydrophilic fragment of TMAO as compared to the two acceptor sites of water. However, this effect is not dominant at lower concentrations of TMAO as is clear from FTIR results in Supplementary Fig. 2 where a blue shift by 3 cm^−1^ for O-D stretching is observed only for increase in the concentration more than 1M. The key findings from the current research emerged out at higher concentrations of TMAO are worthy of further discussion.

At higher concentrations of TMAO, there are less available water molecules which guarantee the interactions among TMAO molecules and also with BM surface^52^. As our experimental set up is at physiological pH, BM exposed surface is having net positive charge which may make possible more favourable interaction between TMAO and BM. This electrostatic favourable interactions decrease the free energy of the unfolded state to a large extent, thus, shifting equilibrium towards more denatured state^43^. This electrostatic destabilization is consistent with the thermodynamic parameters, decreased values of T_m_ and **Δ**G_u_ (Table 1). Moreover, in Table 1, decreased **Δ**C_p_ and **Δ**S_m_ values are also emphasizing on the increased SASA in the native state and further also supported by ANS-binding^20^,^21^,^28^. Again, it is also evident from MD simulation results comprising of SASA for higher concentrations (Fig. 11a). A large number of TMAO molecules bind to the protein surface through hydrogen bonding^56^, thus, intramolecular H-bonding in native form of BM is decreased which can also be depicted from CD results (Fig. 2a), **Δ**H_m_ (Table 1) and also from Fig. 11b for MD simulations. At higher concentrations, TMAO molecules start to enter and bind into the hydrophobic pockets as well ascribing decreased hydrophobic interactions (Fig. 10a,b).

On the basis of the above results and discussions, it is certain that the principle of preferential exclusion/inclusion regarding stabilization/destabilization of protein is not violated at all studied concentration of TMAO. However, an important aspect of this work is to put the plausible mechanism of less obvious behaviour of TMAO i.e. destabilization of protein. The identification of strength of water-water, TMAO-water, protein-water and protein-TMAO hydrogen bonding is clear example of the strength of combining experimental and MD simulation method. This study has shown surprising results which, to our knowledge, has never been reported before. It is clear that this approach can be extended to study the osmolytes mixtures and which can be further utilized by the cell to regulate protein folding and activity. Further efforts are required to find whether TMAO will counteract the high temperature and pressure effects when there is net positive charge on the protein under study and to study counteraction of destabilization effect of TMAO.

## Methods

### Reagents and sample preparations

Bromelain (E.C. 3.4.22.32) lot No. B4882 from Ananas comosus, TMAO, D_2_O, acrylamide and ANS were purchased from Sigma Aldrich, USA. Anhydrous sodium phosphate monobasic and sodium phosphate dibasic dihydrate of highest purity and analytical grade purchased from Sisco Research Lab (SRL), India. For the study of proteolytic activity, casein (Hammarsten) was also obtained from the SRL, India. All other chemicals used were of analytical grade with highest purity. The enzyme samples were prepared in 0.2 M sodium phosphate buffer at pH 7 with 0.5 mg/mL concentration of BM for all measurements. For all gravimetric measurements, Mettler Toledo balance with a precision of ±0.0001 g was used. All mixture samples were prepared using distilled deionized water with resistivity of 18.3 Ωcm. After completely dissolving the enzyme in the solution, all samples were filtered with 0.22 μm disposable filter (Millipore, Millex-GS) through syringe and were incubated for 15 min at 25 °C in order to obtain complete equilibrium before performing experiments. The stability and activity of BM were studied in absence and presence of 0.1, 0.5, 1.0, 1.5, 2.0, 2.5 and 3.0 M of TMAO for all experiments.

### Thermal equilibrium unfolding of BM in the presence of TMAO

TMAO induced equilibrium unfolding studies of the BM were conducted over a temperature range from 15 to 90 °C, using Trp fluorescence as a probe. These thermal denaturation studies were carried out in Cary Eclipse fluorescence spectrofluorimeter (Varian optical spectroscopy instruments, Mulgrave, Victoria, Australia) equipped with an intense Xenon flash lamp as the light source. This spectrometer isequipped with a Peltier-type temperature controller with a precision of ±0.05 °C at a heating rate of 1 °C min^−1^ providing adequate time for equilibration. An excitation wavelength of 295 nmwasused in order to avoid the radiation energy transfer from other fluorescent residues to the Trp residues for the overall fluorescence emission. All unfolding transitions of BM at pH 7.0 were analyzed by assuming this small globular protein closely approaching to the two state unfolding mechanism as shown in equation (1)


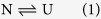


To determine the dependence of intrinsic Trp fluorescence intensity in native and denatured ensembles on temperature, is an essential precondition for extracting the complete thermodynamic profile of protein unfolding. Sigmoidal fluorescence intensity curves were obtained for BM in presence of TMAO (Supplementary Fig. 1). All thermodynamic parameters can be estimated by using Gibbs-Helmholtz equation (2) given below^54^,^55^.


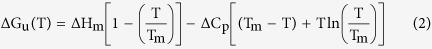


### Steady state fluorescence measurements of BM in varying concentrations of TMAO

Steady state fluorescence emission spectra measurements were carried out at 25 °C in same Cary Eclipse fluorescence spectrofluorimeter using the excitation wavelength at 295 nm. All emission spectra were recorded at a concentration 0.5 mg/mL of BM in presence of a range of concentration of TMAO using a slit width of the excitation and emission at 5 nm and 10 nm, respectively, between 310 and 500 nm. All spectra were averaged of three scans. Fluorescence quenching experiments are performed for the same concentration of protein solution in presence of varying concentration of TMAO with the use of acrylamide concentrations, 0.1 and 0.25 M. Other parameters were kept same. For ANS binding fluorescence experiment, excitation wavelength was set at 380 nm and emission spectra were taken from 385–550 nm.

### Spectral characterization of BM in TMAO using CD spectroscopy

All CD spectral studies were performed for BM with a concentration of 0.5 mg/mL at 25 °C as a function of concentration of TMAO after pre-equilibration of all samples at the 25 °C for 15 min. The CD spectra were recorded using PiStar-180 spectrophotometer (Applied Photophysics, U.K.) equipped with a peltier system for temperature control with an accuracy of ±0.1 °C. The system was calibrated using (1S)-(+)-10-camphorsulfonic acid (Aldrich, Milwaukee, WI), which exhibits a 34.5 M cm^−1^ molar extinction coefficient at 285 nm and 2.36 M cm^−1^ molar ellipticity (Θ) at 295 nm. Far-UV and near-UV CD spectra were monitored in the range 200–250 nm and 250–300 nm in cuvette with pathlength of 0.1 cm and 1 cm, respectively, at a response time of 1 s and 1 nm band width using scan speed 50 nm/min. All spectra were averaged of three scans.

### Activity measurements of BM in TMAO

BM activity was assayed using UV-vis spectrophotometer (UV-1800 Simadzu Spectrophotometer) providing denatured casein solution (0.5 mL of 0.5%) as a substrate which was incubated for 10 min at 25 °C with BM in buffer at pH 7.0 pretreated with the various concentrations of TMAO. Trichloroacetic acid (TCA), 1mL of 110 mM, was used to stop the reaction after 10 min and the precipitates of the undigested casein were removed by the centrifugating the samples. Reaction product was correlated with the absorbance values of a reagent blank which are spectrophotometrically measured at 275 nm. From the standard curve of absorbance of known quantities of the tyrosine (Tyr), activity (units per mL) of BM samples can be determined which is the amount of the micromoles of Tyr equivalents released from casein per min. The activities (units/mL) were calculated using the following equation:


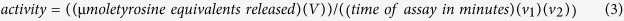


where *V* is total volume of assay in mL, *v*_1_ is volume of enzyme used in mL, *v*_2_ is volume of sample in mL used for UV measurements.

### FTIR characterization of BM in the presence of TMAO

Infrared spectra of stem bromelain in the absence and presence of various concentrations of TMAO were recorded by using a thermo scientific FTIR spectrometer. Samples were held in an IR cells with ZnSe windows and 15 μm path length spacers. 256 scans were performed at 4 cm^−1^ resolution and averaged. All samples were prepared in D_2_O buffer maintained at pD ~7.0 with a fixed concentration of the enzyme and equilibrated for 3–4 hrs to facilitate H-D exchange in the protein sample. To be able to compare the effect of varying concentration of the TMAO, a proper subtraction of D_2_O buffer was carried out.

### FTIR bands analysis

To quantify the IR spectra, curve fitting were performed for original IR spectra of each and every sample using omnic software to estimate number, position, band shapes, widths and heights of the bands. The curve fitting was executed without smoothed spectra. The initial parameters were iterated using a combination of Gaussian and Lorentzian functions with a constant baseline. An iterative process was followed in two steps; (i) band positions were fixed and other parameters were iterated, (ii) all parameters were iterated. Typically, band positions are shifted by less than 1 cm^−1^ in curve fitting procedure from their actual positions. The number of bands is also found to be changed relatively small during curve fitting. There is little uncertainty in the integrated intensities of the individual bands because of not fixing of band position and band width in curve fitting process.

### MD simulation studies of BM at different concentrations of TMAO

To gain atomistic level insight into the dynamics of BM as a function of different concentrations of TMAO, Molecular Dynamics (MD) studies were carried out. Structure of BM was obtained from RCSB^57^ model (PDBID: 1W0Q). MD simulation was performed using AMBER v. 14^58^,^59^ suite on Nvidia K20 GPU cards. Protein and TMAO were solvated in an octahedral box of TIP3P^60^ water molecules. System was neutralized using counter ions. Periodic boundary conditions were applied throughout the system with PME summation^61^ for electrostatic calculations. Covalent bonds to hydrogen atoms were restricted using SHAKE methodology^62^. Berenson thermostat^62^ was utilized with constant pressure conditions. 2 fs time-steps along with 8 Angstrom cutoff for non-bonded interactions were applied for the MD studies. System was energy minimized using 250 step Steepest Decent followed by 750 steps of Conjugant Gradient. Water and TMAO mixture was heated to 300 K gradually, keeping the solute protein fixed with a force of 25 N. Slowly restraints were removed step wise till 0.1 N and fully unrestrained system was heated at 300K. Systems were equilibrated for another 5 ns at 300 K to attain stability. Convergence of energy and density was monitored. Production phase simulations were carried out for 250 ns using BM, explicit solvent and varying concentrations of TMAO. All MD simulations were carried out on GPU cluster at Supercomputing Facility for Bioinformatics and Computational Biology (SCFBio), IIT Delhi, India.

## Additional Information

**How to cite this article**: Rani, A. *et al.* Trimethylamine-*N*-oxide switches from stabilizing nature: A mechanistic outlook through experimental techniques and molecular dynamics simulation. *Sci. Rep.* **6**, 23656; doi: 10.1038/srep23656 (2016).

## Supplementary Material

Supplementary Information

## Figures and Tables

**Table 1:** The thermodynamic parameters determined by the fluorescence analysis of thermal denaturation of BM in absence and presence of varying concentrations of TMAO; Transition temperature (T_m_), Gibbs free energy change of unfolding (ΔG_u_) at 25 °C, enthalpy change of unfolding (ΔH_m_) at T_m_, entropy change of unfolding (ΔS_m_) at T_m_ and heat capacity change of unfolding (ΔC_p_) at 25 °C.

Concentration of TMAO (M)	T_m_(°C)	ΔG_u_ (kcal/mol)	ΔH_m_ (kcal/mol)	ΔS_m_ (kcal/mol/K)	ΔC_p_ (kcal/mol/K)
0.0	65.5	4.07	22.3	0.065	0.455
0.1	66.6	4.13	22.7	0.067	0.479
0.5	67.2	4.24	23.6	0.070	0.516
1.0	67.9	3.85	24.4	0.072	0.552
1.5	65.9	3.76	17.5	0.052	0.232
2.0	65.1	3.64	15.5	0.046	0.131
2.5	64.6	3.43	13.6	0.040	0.034
3.0	63.3	3.02	12.0	0.036	−0.064

The error in Tm does not exceed 0.1 °C. The estimated relative uncertainties in ΔG_u_, ΔH_m_ and ΔS_m_ are around 2% of the reported values. All values are averaged of three concordant readings.
