# Supplementary material for: Trimethylamine-N-oxide switches from stabilizing nature: A mechanistic outlook through experimental techniques and molecular dynamics simulation
Source: Sci Rep. 2016 Mar 30;6:23656. doi: 10.1038/srep23656 (PMC4812290; doi:10.1038/srep23656)
Supplement: Supplementary Information [file srep23656-s1.pdf]

Supplementary Information

Trimethylamine-*N*-oxide switches from stabilizing nature: A mechanistic outlook through experimental techniques and molecular dynamics simulation

Anjeeta Rani[1], Abhilash Jayaraj[2], B. Jayaram[2] and Pannuru Venkatesu*[1]

[1]Department of Chemistry, University of Delhi, Delhi – 110 007, India

[2]Department of Chemistry, Indian Institute of Technology, New Delhi - 110 016, India

Thermal equilibrium unfolding of BM in the presence of varying concentrations of TMAO by using fluorescence spectroscopy.

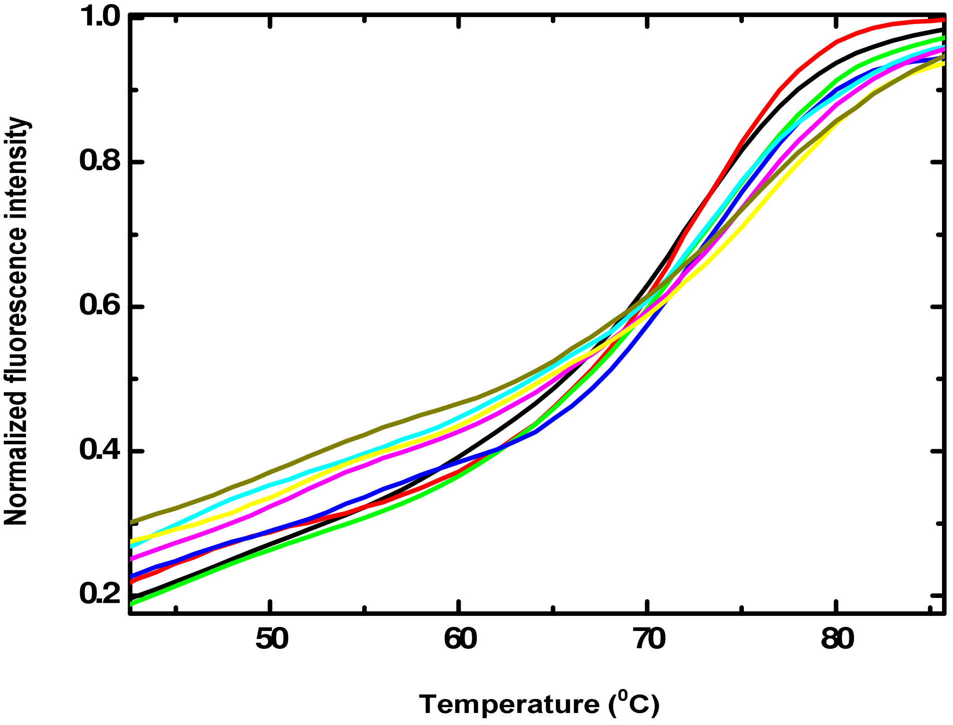

Supplementary Figure 1| Thermal fluorescence intensities spectra as a function of temperature for BM in absence (black) and presence of varying concentrations of TMAO; 0.1 M (red), 0.5 M (green), 1.0 M (blue), 1.5 M (cyan), 2.0 M (pink), 2.5 M (yellow), 3.0 M (dark yellow).

FTIR characterization of structural changes in BM in the presence of TMAO.

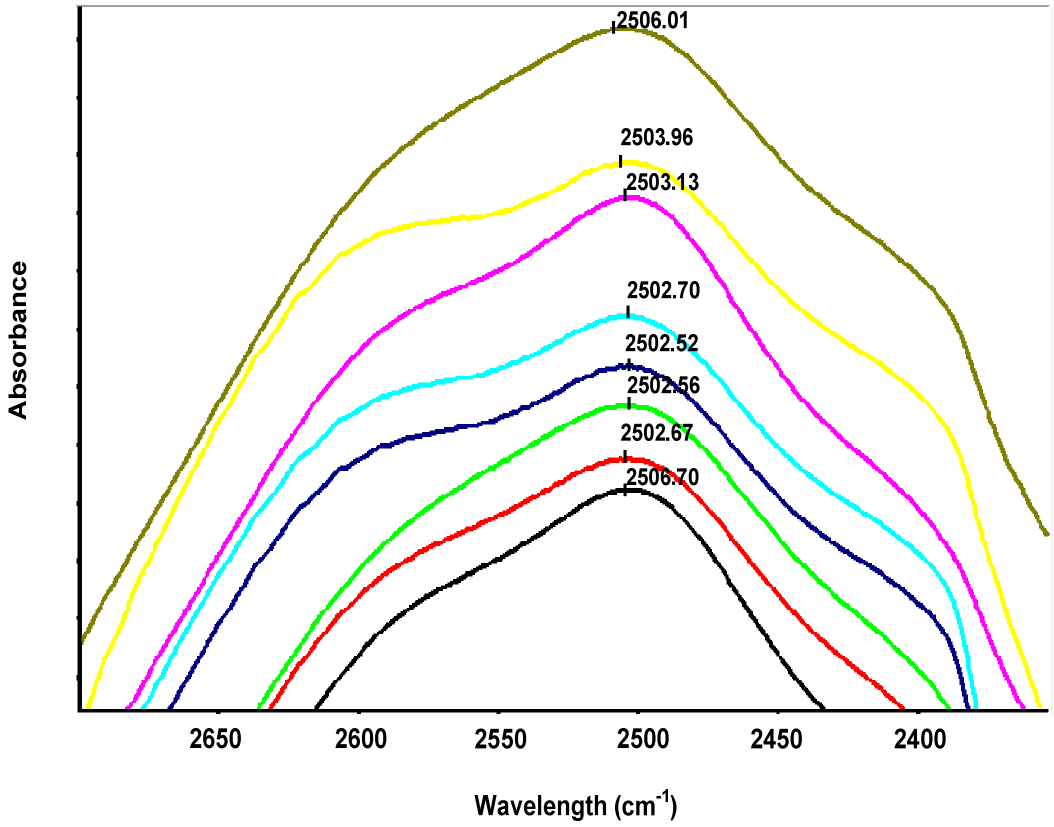

Supplementary Figure 2| FTIR stacked spectra showing O-D stretching band of D_2O at 25^0C for solutions of BM in absence (black) and presence of varying concentrations of TMAO; 0.1 (red), 0.5 (green), 1.0 (blue), 1.5 (cyan), 2.0 (pink), 2.5 (yellow) and 3.0 M (dark yellow).

FTIR characterization of structural changes in BM in the presence of TMAO.

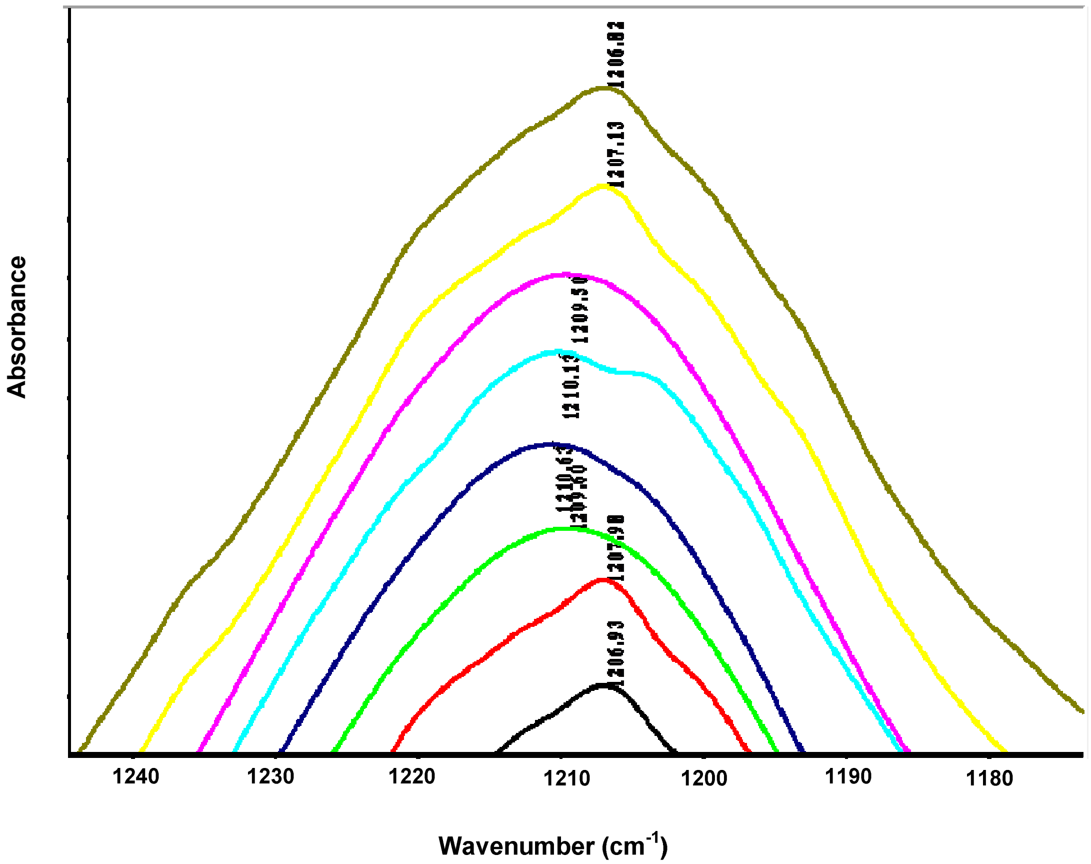

Supplementary Figure 3| FTIR stacked spectra showing O-D bending frequencies of D_2O at 25^0C for solutions of BM in absence (black) and presence of varying concentrations of TMAO; 0.1 (red), 0.5 (green), 1.0 (blue), 1.5 (cyan), 2.0 (pink), 2.5 (yellow) and 3.0 M (dark yellow).

Supplementary Table 1| Bands assignment and respective integrated intensities for the curve fitted original FTIR spectra of BM in D_2O at 25 ^{0}C. Band positions are shifted by less than 1 cm^{-1} from their actual positions during curve fitting procedure. The integrated intensities of individual bands have uncertainties ≤ 1% for three concordant measurements.

BM in D_2O at pD 7 (25 ^{0}C)		
Band position	% area	assignment
1624	0.2	Extended beta
1629	5.3	beta
1632	3.7	beta
1637	0.2	beta
1640	11.3	Beta sheet
1643	1.9	Solvent exposed helix
1647	9.2	Random coils
1651	8.7	Alpha helix
1656	3.1	Alpha helix
1659	10.4	Alpha helix
1667	23.6	Turn, bend, loops
1674	1.2	Anti beta sheet
1678	10	turn
1683	7.5	turn
1694	3.5	turn

Supplementary Table 2 | Secondary structure contents of BM in absence and presence of varying concentrations of TMAO from FTIR curve fitted original spectra analysis. The integrated intensities of individual bands have uncertainties ≤ 1% for three concordant measurements.

Concentration of TMAO [M]	Alpha %	Beta %	Turn, loops and bends %	Random %	Total (α + β) characters (%)
0.0	24	22	45	9	46
0.1	18	32	39	11	50
0.5	26	30	36	8	56
1.0	29	29	34	8	58
1.5	27	25	43	5	52
2.0	25	22	46	7	46
2.5	19	20	52	9	39
3.0	14	20	54	12	34
